# Three comments on the methodology of a hypothermia study

**DOI:** 10.1002/jgf2.434

**Published:** 2021-03-15

**Authors:** Yuki Tachibana, Kazuoki Inoue, Shin Yoshida, Kentaro Kinjo, Daisuke Kato, Yuki Kataoka

**Affiliations:** ^1^ Department of General Medicine Okinawa Prefectural Yaeyama Hospital Okinawa Japan; ^2^ Department of Community‐based Family Medicine School of Medicine Tottori University Faculty of Medicine Yonago Japan; ^3^ Kaita Hospital Fukuoka Japan; ^4^ Department of General Medicine Emergency Medicine Teikyo University School of Medicine Itabashi‐ku Japan; ^5^ Department of Family Medicine Mie University Graduate School of Medicine Tsu Japan; ^6^ Department of Respiratory Medicine Hyogo Prefectural Amagasaki General Medical Center Amagasaki Japan

To the Editor,

With great interest, we read this study, which identified predictors of mortality caused by hypothermia in Japan^1^. The prognostic factors of hypothermia in Japan are unknown, and the high incidence and mortality of the condition in the elderly have prompted this study^2^. However, we have three concerns about its methodology.

First, the study included patients who had a cardiac arrest and those who died in the emergency room (ER), which may be reverse causality. In other words, cardiac arrest or death shortly after entering the ER may have resulted in hypothermia. This inclusion criterion would increase the C‐statistics of the in‐hospital mortality prediction score because the near‐death state would contribute not only to hypothermia but also to lower arterial pressure and higher creatinine levels.

Second, the study did not state to what extent the C‐statistics changed upon adjusting the cutoff value and reducing the number of adjustment variables from five to three. The accuracy of these three adjustment variables before altering the cutoff values^3^ and, if possible, the results of the analysis after adjusting for optimism should be included in the study^4^ to provide useful information to the readers.

Finally, the small sample size may be a statistical limitation. We are concerned about the possibility of overfitting by using five variables. In other words, since the main outcome was the 17 deaths, conducting logistic regression analysis using multiple explanatory variables may be inappropriate^5^.

We believe that the in‐hospital mortality score arrived at in this study will be more useful to several hospitalists if these three perspectives are considered.

## CONFLICT OF INTEREST

The authors have stated explicitly that there are no conflicts of interest in connection with this article.
